# 24-hour powerful blood pressure lowering – essential for target organ protection

**Published:** 2010-02

## Introduction

Circadian blood pressure rhythm is controlled by intracellular molecular clocks, which allow the body to prepare for anticipated stimuli, the morning blood pressure surge helping to meet the challenges of the day while the nocturnal blood pressure fall sets the system for a period of rest.

**Fig. 1.**
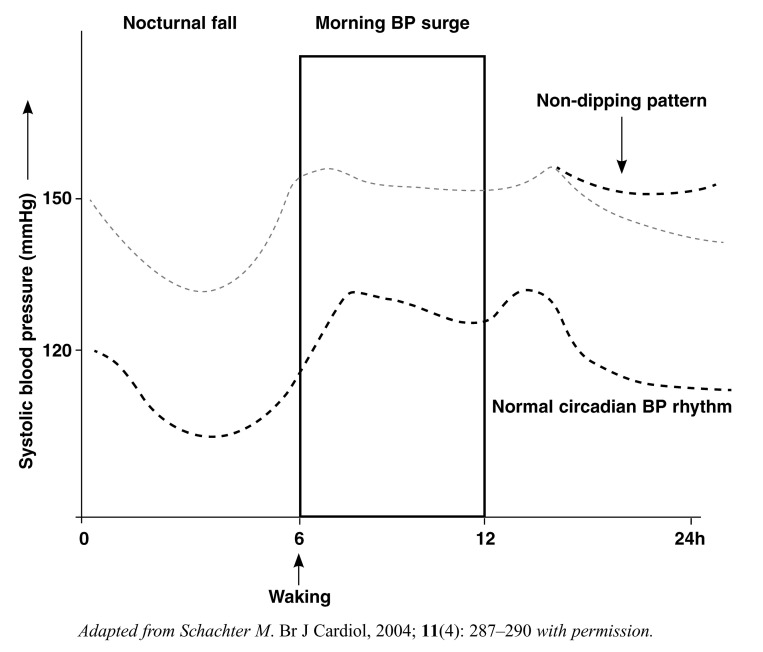
Adapted from Schachter M. Br J Cardiol, 2004; 11(4): 287–290 with permission. Circadian variations in hypertensive (top curve) and normotensive (lower curve) individuals.

These rhythms vary in hypertensive and non-hypertensive individuals (Fig. 1).^1^ Cardiovascular outcomes are worsened in individuals who have an excessive morning blood pressure surge and in those who lack the normal nocturnal blood pressure fall.

This early morning blood pressure surge has also been shown in epidemiological studies to result in a clustering of cardiovascular complications such as stroke,^2^ myocardial infarction3 and coronary ischaemia^4^ around this morning period. This increased risk of cardiovascular events has been shown to occur also in elderly hypertensives.^5^

Early morning blood pressure surges occur in both poorly controlled and well-controlled hypertensives; with poor blood pressure control being associated with uncontrolled morning blood pressure levels in 70% of patients in the ACAMPA study (Analysis of the Control of blood pressure using Ambulatory Blood Pressure monitoring).^6^

Few antihypertensives are sufficiently long acting to sustain adequate blood pressure lowering for the full 24 hours between once-daily doses, and indeed many are at their lowest efficacy during the risky early morning period. The angiotensin receptor blocker (ARB) telmisartan has the longest plasma half-life, highest lipophilicity, highest receptor binding affinity, and slowest dissociation of any ARB, making it particularly suitable for sustained 24-hour blood pressure control.

An important measure of 24-hour blood pressure control is the smoothness index (SI), which is considered to be a better indicator of blood pressure homogeneity over time.7 The SI is calculated by using the average of hourly changes in blood pressure over 24 hours, divided by the standard deviation. SI values greater than one indicate a reliable and sustainable effect over 24 hours.

A recent meta-analysis,^8^ which assessed the SI for standard daily doses of drugs from different classes showed that telmisartan has an SI higher than that of losartan, valsartan or ramipril, and equivalent to the SI of the long-acting antihypertensive amlodipine.

The predictive value of telmisartan’s SI of 1.13 systolic blood pressure (SBP) and 0.97 diastolic blood pressure (DBP) and the provision of 24-hour protection is supported by the results of two major trials, the MICADO II study^9^ and PRISMA I and II studies.^10^,^11^ The MICADO II study showed telmisartan (80 mg) has a more powerful SBP reduction throughout the 24-hour period compared to valsartan (160 mg; recommended dose).

In the PRISMA I and II studies (Prospective, Randomized Investigation of the Safety and efficacy of telmisartan vs ramipril using Ambulatory BP Monitoring), in which patients with mild-to-moderate hypertension (95–109 mmHg seated DBP) were randomised to once-daily treatment with telmisartan 80 mg or ramipril 5 mg force-titrated to 10 mg, researchers found that the reductions in both SBP and DBP during the last six hours of the dosing interval were significantly greater with telmisartan 80 mg than with ramipril 10 mg (p < 0.0001). Similar findings were observed throughout the 24-hour dosing period.

Prescribing antihypertensives such as telmisartan with a prolonged duration of action not only provides blood pressure lowering during the vulnerable early morning hours but also mitigates against the loss of blood pressure control if a dose is missed. In a pooled analysis of two studies comparing telmisartan 80 mg with valsartan 160 mg once daily during the 24 hours after a missed dose in patients with mild-to-moderate hypertension (MICADO I and II studies), telmisartan sustained blood pressure control after a missed dose significantly better than with valsartan (p < 0.05 daytime; p < 0.001 night time), an effect that was particularly marked during the last six hours of the dosing interval (p < 0.0001).

In conclusion, clinical studies have shown that telmisartan provides 24-hour blood pressure control superior to that of the ARBs, losartan and valsartan, the calcium-channel blocker, amlodipine, and the ACE inhibitor, ramipril. This agent is particularly effective during the last six hours of the dosing interval when other antihypertensives tend to have reduced effectiveness. Telmisartan is, therefore, a highly appropriate antihypertensive for sustained 24-hour blood pressure control, especially during the risky early morning hours.
